# Deficiency of PI3-Kinase catalytic isoforms p110γ and p110δ in mice enhances the IL-17/G-CSF axis and induces neutrophilia

**DOI:** 10.1186/s12964-017-0185-y

**Published:** 2017-07-19

**Authors:** Kirsten Bucher, Fee Schmitt, Benedikt Mothes, Carolin Blumendeller, Daniel Schäll, Roland Piekorz, Emilio Hirsch, Bernd Nürnberg, Sandra Beer-Hammer

**Affiliations:** 1 Department of Pharmacology and Experimental Therapy, Institute of Experimental and Clinical Pharmacology and Toxicology and ICePhA mouse clinic, University of Tübingen, D-72074 Tübingen, Germany; 2 Institute of Biochemistry and Molecular Biology II, University of Düsseldorf, D-40225 Düsseldorf, Germany; 3 Department of Genetics, Biology and Biochemistry, University of Torino, I-10126 Torino, Italy; 4 Institute of Experimental and Clinical Pharmacology and Toxicology, University Tübingen, Wilhelmstraße 56, D-72074 Tübingen, Germany

**Keywords:** PI3K p110γ, PI3K p110δ,neutrophil homeostasis, IL-17-producing T cells, Il-17, G-CSF

## Abstract

**Background:**

Phosphoinositide 3-kinase γ (PI3Kγ) and PI3Kδ are second messenger-generating enzymes with key roles in proliferation, differentiation, survival, and function of leukocytes. Deficiency of the catalytic subunits p110γ and p110δ of PI3Kγ and PI3Kδ in p110γ/δ^−/−^ mice leads to defective B- and T-cell homeostasis. Here we examined the role of p110γ and p110δ in the homeostasis of neutrophils by analyzing p110γ^−/−^, p110δ^−/−^ and p110γ/δ^−/−^ mice.

**Methods:**

Neutrophils and T cells in leukocyte suspensions from the bone marrow (BM), blood, spleen and lung were analyzed by flow cytometry. Serum concentrations of IL-17, of the neutrophilic growth factor G-CSF, and of the neutrophil mobilizing CXC chemokines CXCL1/KC and CXCL2/MIP-2 were measured by Bio-Plex assay. Production of G-CSF and CXCL1/KC by IL-17-stimulated primary lung tissue cells were determined by ELISA, whereas IL-17-dependent signaling in lung tissue cells was analyzed by measuring Akt phosphorylation using immunoblot.

**Results:**

We found that in contrast to single knock-out mice, p110γ/δ^−/−^ mice exhibited significantly elevated neutrophil counts in blood, spleen, and lung. Increased granulocytic differentiation stages in the bone marrow of p110γ/δ^−/−^ mice were paralleled by increased serum concentrations of G-CSF, CXCL1/KC, and CXCL2/MIP-2. As IL-17 induces neutrophilia via the induction of G-CSF and CXC chemokines, we measured IL-17 and IL-17-producing T cells. IL-17 serum concentrations and frequencies of IL-17^+^ splenic T cells were significantly increased in p110γ/δ^−/−^ mice. Moreover, IFN-γ^+^, IL-4^+^, and IL-5^+^ T cell subsets were drastically increased in p110γ/δ^−/−^ mice, suggesting that IL-17^+^ T cells were up-regulated in the context of a general percentage increase of other cytokine producing T cell subsets.

**Conclusions:**

We found that p110γ/δ deficiency in mice induces complex immunological changes, which might in concert contribute to neutrophilia. These findings emphasize a crucial but indirect role of both p110γ and p110δ in the regulation of neutrophil homeostasis.

**Electronic supplementary material:**

The online version of this article (doi:10.1186/s12964-017-0185-y) contains supplementary material, which is available to authorized users.

## Background

Neutrophil granulocytes are the most abundant circulating phagocytes and serve as first line of innate immune defense in microbial infections. They produce a wide range of antimicrobial effectors including reactive oxygen species like superoxide radicals and a variety of antibacterial proteins. The homeostasis of neutrophils needs to be tightly regulated, as acquired or idiopathic neutropenia predisposes to infections, whereas activation and accumulation of increased numbers of neutrophils can promote tissue damage in inflammatory disorders, such as severe asthma, COPD or rheumatoid arthritis [1, 2]. Additionally, elevated neutrophil counts have been shown to be an independent risk factor for cardiovascular morbidity and cancer-related mortality (references in [1]). Numbers of circulating neutrophils are regulated by various mechanisms, including the generation and storage of neutrophils in the bone marrow (BM), their release into the blood, and the clearance of apoptotic neutrophils [2]. The most important growth factor for neutrophils is G-CSF, which directly regulates their proliferation and differentiation [3, 4]. The pro-inflammatory cytokine IL-17 signals up-stream of G-CSF and drives G-CSF production and neutrophilia [5]. Cellular sources of IL-17 are IL-17-producing CD4^+^ T helper cells (Th17) [6], some CD8^+^ T cells [7], and various innate immune cell types [8].

Heterodimeric PI3Kγ and PI3Kδ are cell surface receptor-triggered signal transducing enzymes which belong to class I PI3Ks. The catalytic class IA subunits p110α, p110β, and p110δ couple with the regulatory subunit p85, whereas the only catalytic class IB subunit p110γ binds to one of two regulatory subunits p87/p84 or p101 [9]. PI3Kγ and PI3Kδ are activated by partially overlapping pathways [9]. Thus, PI3Kγ is mainly responsive to G protein-coupled receptors such as chemokine receptors, and is stimulated by Gβγ and Ras [9]. In contrast, PI3Kδ is typically activated by receptor tyrosine kinases, cytokine receptors, B- and T-cell receptors, Toll-like receptors, antigen receptors, and also by Ras [9–11]. Upon activation, PI3Kγ and PI3Kδ generate phosphatidylinositol-3,4,5-trisphosphate (PIP_3_) within the inner leaflet of the plasma membrane. PIP_3_ serves as a binding site for downstream signaling proteins, like the serine/threonine kinase Akt and PDK1. Activation of these kinases mediates various cellular responses ranging from cell proliferation to cell motility [12].

The catalytic subunits p110γ and p110δ are preferentially expressed in leukocytes. Accordingly, p110γ or p110δ knock-out or kinase-dead knock-in mice exhibit various immunological changes [12]. Studies investigating p110γ or p110δ in neutrophil responses showed that p110γ plays a role in chemokine-induced Akt phosphorylation, respiratory burst, and migration [10, 13], whereas p110δ is required for neutrophil movement to chemotactic stimuli [14]. Moreover, both isoforms have temporally distinct roles in chemokine-induced neutrophil migration [15].

IL-17 can induce G-CSF-dependent neutrophilia and interestingly, p110γ and p110δ have also been shown to regulate IL-17 responses. Thus, pharmacological inhibition of p110δ or genetic inactivation of the catalytic activity of either p110γ or p110δ reduces IL-17 levels and/or numbers of IL-17-producing T cells in mouse models of asthma [16], psoriasis [17], or multiple sclerosis [18]. Moreover, p110γ [19] or p110δ [20] have been implicated in Th17 cell differentiation [19, 20] and intracellular IL-17 signaling [21].

We have previously demonstrated that B-cell development and maintenance is compromised in p110γ/δ^−/−^mice [22]. During that study we also observed a striking infiltration of CD11b^+^ Gr-1^hi^ neutrophils in the spleens of p110γ/δ^−/−^mice. Here we investigated the potential mechanisms leading to increased neutrophil levels in p110γ/δ^−/−^mice. To this end, we analyzed the cell death rate of neutrophils, the differentiation of granulocytes, neutrophil homing, the cytokine production of neutrophilic growth and release factors, and the potential cellular sources of these immune mediators in WT, p110γ^−/−^, p110δ^−/−^, and p110γ/δ^−/−^ mice.

## Methods

### Mice

Wild-type (WT), p110γ^−/−^, p110δ^−/−^ and p110γ/δ^−/−^ mice on a C57BL/6 N genetic background were bred as described previously [10, 22–24]. For experiments 10–14 week old male and female mice were used.

### Preparation of leukocyte suspensions from BM, blood, spleen and lung

Single cell suspensions from blood, spleen, lungs and BM were isolated as described previously [22, 24, 25, 26]. Leukocytes from single cell suspensions were prepared by lysis of red blood cells in ACK buffer (0.155 M NH_4_Cl, 0.01 M KHCO_3_, 0.1 mM EDTA). Total cell counts were determined with a Neubauer chamber with trypan blue exclusion of dead cells.

### Flow cytometry

For flow cytometric characterization of cells, the following antibodies, peptides or reagents were used: 7-aminoactinomycin D (7-AAD), CD3ε-APC (1:100; AB_398529; clone 145-2C11), CD3ε-Pacific Blue (1:100; AB_397063; clone 500A2), CD3ε-PerCPCy5.5 (1:25; AB_10562558, clone 145-2C11), CD4-APC.Cy7 (1:400; AB_398529; clone GK1.5), CD4-PE.Cy7 (1:100; AB_394461; clone RM4–5), CD11b-PE.Cy7 (1:400; AB_394491; clone M1/70), CD16/32 (1:50; AB_394657; 2.4G2), CD19-V450 (1:200; AB_1645270; clone 1D3), CD182(CXCR2)-APC (1:400; FAB2164A, clone 242,216), CD184(CXCR4)-APC (1:400; AB_1645219; clone 2B11), Gr-1-PE (1:400; AB_394644; clone RB6-8C5), IL-4-APC (1:100; AB_398556; clone 11B11), Ly6G-APC (1:200; AB_1727560; clone 1A8), Ly6G-FITC (1:200; AB_394207; clone 1A8) and Ly6G-PE (1:200; AB_394208; clone 1A8), Siglec-F-PE (1:200; AB_394341; clone E50–2440) (all BD Biosciences, Heidelberg, Germany), IL-5-Brilliant Violet 421 (1:80; AB_2563161; clone TRFK5), IL-17A-PE (1:80; AB_315464; clone TC11-18H10.1), NK1.1-Brilliant Violet 510 (1:20; AB_2562216; clone PK136), NK1.1-PerCPCy5.5 (1:100; AB_2132705; clone PK136) (all Biolegend, San Diego, USA), IFN-γ-FITC (1:100; AB_465411; clone XMG1.2) (eBiosciences, San Diego, CA, USA), and CD8a-Vioblue (1:20; AB_10829751; clone 53–6.7; Miltenyi, Bergisch Gladbach, Germany). To analyze the intracellular expression of cytokines in T cells, splenocytes were incubated with 50 ng/mL phorbol 12-myristate 13-acetate (PMA), 1 μg/mL ionomycin, and 5 μg/mL Brefeldin A (all Sigma-Aldrich, Taufkirchen, Germany) in cell culture medium (RPMI supplemented with 10% FCS, 2 mM glutamine, 0.05 mM β-mercaptoethanol, and 100 U/mL each of penicillin and streptomycin; all PAA, Pasching, Austria) at 37 °C for 3.5 h. Cells were harvested and incubated with Live/Dead Fixable Aqua Dead Cell Stain (Invitrogen, GmbH, Karlsruhe, Germany) to exclude dead cells. Then, cells were surface stained for 15 min at 4 °C. Thereafter, they were fixed and permeabilized followed by intracellular cytokine staining using a Transcription Factor Buffer Set according to the manufacturer’s protocol (BD Biosciences, Heidelberg, Germany). Flow cytometry measurements were performed on a FACSCantoTM II (BD Biosciences) and data were evaluated with FlowJo software.

### Adoptive transfer of anti-Ly6G-labeled BM-leukocytes

The homing of adoptively transferred “young” and “senescent” neutrophils was analyzed in WT and p110γ/δ^−/−^ mice using a refined method described by Martin et al.*,* [27]. In brief, equal fractions of “senescent” neutrophils contained in BM cell suspensions that had been incubated for 6 h in vitro (6 h neutrophils), and “young” neutrophils, contained in freshly isolated bone marrow (0 h neutrophils), were prepared. Then, BM cells were stained with anti-Ly6G (1A8)-APC (6 h neutrophils) or anti-Ly6G (1A8)-PE (0 h neutrophils). APC- and PE-coupled Ly6G antibodies were chosen since neutrophils labeled with these antibodies showed comparable retrieval rates following adoptive transfer into mice [25]. After washing twice with PBS, the separately stained anti-Ly6G (1A8)-APC^+^ and -PE^+^ BM-leukocytes were pooled and 2.5 × 10^7^ cells in 150 μL PBS were injected into the tail vein of each recipient mouse. WT 0 h and 6 h BM cells were injected into WT mice, whereas p110γ/δ^−/−^ 0 h and 6 h BM cells were injected into p110γ/δ^−/−^ mice respectively. After one hour, leukocyte suspensions from BM, blood, and spleen of recipients were prepared. Cells were counted and stained with antibodies against CD3ε, CD11b, and CD19 and analyzed using flow cytometry. Myeloid cells were analyzed for the presence of 1A8-APC- or -PE-labeled neutrophils. In each experiment, percentages of APC- and PE-labeled neutrophils were also determined in an aliquot of the injection suspension. Retrieved cells were calculated as percentages of injected Ly6G (1A8)-labeled cells**.**

### Cultivation of primary lung tissue cells

Single cell suspensions from lungs were incubated in DMEM (Sigma-Aldrich, Taufkirchen, Germany) supplemented with 20% FCS, 2 mM glutamine, 0.05 mM β-mercaptoethanol, and 100 U/mL each of penicillin and streptomycin (all PAA, Pasching, Austria). On day 2, non-adherent cells were washed off using PBS and medium was exchanged. On day 6, cells were detached with trypsin/EDTA (PAA, Pasching, Austria) and split at a ratio of 1:2. Medium was exchanged on day 11 and cells were harvested on day 14. For the analysis of cytokine production, cells were seeded at 10^5^ cells per well into 96-well plates. Following incubation at 5% CO_2_ and 37 °C for 24 h, the medium was removed and IL-17A was added in increasing concentrations. After another 24-h-incubation the supernatant was harvested. For analysis of Akt- and p38-phosphorylation, cells were incubated at 5 × 10^5^ cells per well in 24-well plates. After 24 h the medium was removed and cells were stimulated with 50 ng/mL IL-17A. After 0, 5, 10, 30 and 60 min cells were lysed as described below.

### Isolation and ex vivo stimulation of primary T cells

For analyses of T-cell cytokine production, splenocytes were purified using a Pan T-cell Isolation Kit (Miltenyi, Bergisch Gladbach, Germany) according to manufacturer’s instructions. The purity of CD3ε T cells was >90% as confirmed by flow cytometry. T cells were incubated in 24-well plates pre-coated with plate-bound anti-CD3ε (1 μg/mL or 10 μg/mL, BD Biosciences, Heidelberg, Germany) or anti-CD3ε (1 μg/mL or 10 μg/mL; AB_394591; clone 145-2C11, BD Biosciences, Heidelberg, Germany) or anti-CD3ε (1 μg/mL) / anti-CD28 (10 μg/mL; AB_396676; clone 37.51, BD Biosciences) in cell culture medium (RPMI supplemented with 10% FCS, 2 mM glutamine, 0.05 mM β-mercaptoethanol, and 100 U/mL each of penicillin and streptomycin). After incubation for 48 h the supernatant was harvested.

### Measurement of cytokines

Serum concentrations of 24 different cytokines (Table 1) including IL-17A, G-CSF, CXCL1/KC, and CXCL2/MIP-2 were determined with a Bio-Plex mouse cytokine 23-plex assay, which was complemented by a Bio-Plex MIP-2-plex assay (Bio-Rad Laboratories, Munich, Germany), both according to the manufacturer’s protocol. Cytokines in the supernatants of BM cells, stimulated T cells, or stimulated primary lung tissue cells were determined by sandwich ELISA. Commercial ELISA kits for IL-17A were from Biozol (Biozol, Eching, Germany) and for IL-4, IL-5, G-CSF, CXCL1/KC, CXCL2/MIP-2, and IFN-γ from R&D (R&D Systems, Minneapolis, USA).

**Table 1:** Cytokine concentrations in the serum of WT, p110γ^−/−^, p110δ^−/−^ and p110γ/δ^−/−^ mice

	WT		p110γ^−/−^		p110δ^−/−^		p110γ/δ^−/−^		*P* value		
Cytokine [pg/mL]	Mean	SD	Mean	SD	Mean	SD	Mean	SD	p110γ/δ^−/−^ vs WT	p110γ^−/−^ vs WT	p110δ^−/−^ vs WT
IL-1α	61.66	21.76	52.67	8.498	45.91	11.99	57.16	15.99	ns	ns	ns
IL-1β	740.2	104.1	754.9	186.3	609.3	78.74	748.2	157.3	ns	ns	ns
IL-2	85.63	14.55	106.7	34.1	88.36	22	116	36.2	ns	ns	ns
IL-3	62.09	28.82	59.57	23.28	49.09	10.25	65.89	17.22	ns	ns	ns
IL-4	57.3	12.58	60.4	18.53	51.03	9.578	63.23	15.86	ns	ns	ns
IL-5	99.54	18.23	109.1	21.39	97.76	15.41	140	24.72	*P* < 0.001	ns	ns
IL-6	32.77	3.331	32.01	9.266	28.42	8.041	59.3	49.9	ns	ns	ns
IL-9	1221	333.8	1174	373.5	965.2	275.3	702.9	280.5	*P* < 0.01	ns	ns
IL-10	202.2	88.91	177.2	71.64	219.1	46.9	270	64.87	ns	ns	ns
IL-12p40	408.1	86.52	294.1	60.73	424.1	70.11	988	346.8	*P* < 0.001	ns	ns
IL-12p70	428.1	107.1	423.7	108.2	367.7	58.5	459.9	119.2	ns	ns	ns
IL-13	1380	227.7	1426	325	1141	171.2	1250	237.9	ns	ns	ns
IL-17A	339.7	42.02	343	41.05	330.5	45.58	460.6	126.4	*P* < 0.01	ns	ns
Eotaxin	3300	912.6	4009	1632	2769	954	3215	1206	ns	ns	ns
G-CSF	306.3	91.89	352.9	85.8	268.3	195.9	804.4	610.4	*P* < 0.01	ns	ns
GM-CSF	744.6	68.77	728.5	151.8	666.9	88.77	709.9	108.6	ns	ns	ns
IFNγ	38.5	7.972	39.27	13.17	30.44	8.659	37.96	18.14	ns	ns	ns
KC	17.48	17.54	52.59	34.61	30.36	23.71	77.14	18.33	*P* < 0.001	*P* < 0.05	ns
MCP-1	1243	134.1	1358	284.9	1199	176.9	1572	587.8	ns	ns	ns
MIP-1α	102.7	28.35	93.29	28.18	79.96	24.74	93.43	26.96	ns	ns	ns
MIP-1β	121.4	14.8	134.2	41.05	116.8	22.38	157.8	34.05	ns	ns	ns
MIP-2	35.92	3.688	39.52	8.501	34.94	6.841	50.16	10.04	*P* < 0.01	ns	ns
RANTES	113.8	17.89	115.9	23.29	112.9	32.27	203.7	88.48	*P* < 0.01	ns	ns
TNFα	1127	157.2	1127	253.5	1036	124.5	1219	312.8	ns	ns	ns

*n* = 10 mice per group

### Immunoblotting

Cultured primary lung tissue cells were incubated in lysis buffer (50 mM Tris, 150 mM NaCl, 2 mM EDTA, 1% NP-40), freshly supplemented with protease and phosphatase inhibitors (PhosSTOP and cOmplete Mini, Roche, Mannheim, Germany) for 30 min on ice. Lysates were cleared by centrifugation. The protein content was measured with the DC Protein Assay (Bio-Rad, Hercules, CA, USA). Then lysates were fractioned by SDS/PAGE (12% acrylamide; Roth, Karlsruhe, Germany), and proteins were transferred to Protran nitrocellulose membranes (GE Healthcare, Freiburg, Germany). Primary antibodies used for immunoblotting were pan-Akt (AB_915783; clone C67E7), phospho-Akt (Ser473) (AB_2315049; clone S473/D9E), p110α (AB_2165248; clone C73F8), p110β (AB_2165246; clone C33D4), p110γ (AB_1904087; clone D55D5), p110δ (D1Q7R), p38 (AB_9212S; polyclonal), phospho-p38 (T180/Y182) (AB_4511S; clone D3F9), tubulin (sc-5286; clone B-7), and GAPDH (AB_561053; clone 14C10) followed by incubation with HRP-conjugated anti-rabbit IgG as secondary antibody (AB_2099233) (all antibodies 1:1000 and all from Cell Signaling Technology, Danvers, MA, USA; except tubulin was from Santa Cruz, Dallas, Texas, USA). Immunoreactive bands were visualized using ECL chemiluminescence system (GE Healthcare, Freiburg, Germany) or SuperSignal West Femto Chemiluminescent Substrate (Thermo Scientific, Rockford, IL, USA). Signals were acquired with a Versa Doc 4000 MP imaging system (Bio-Rad, Hercules, CA, USA).

### Statistical analysis

Statistical analyses were performed as indicated in the figure legends. All calculations were performed using GraphPad Prism 5.01 (GraphPad Software, La Jolla CA, USA). A value of *P* ≤ 0.05 was considered statistically significant.

## Results

### p110γ/δ deficiency in mice increases neutrophil numbers in blood, spleen, and lung

**Fig. 1:**
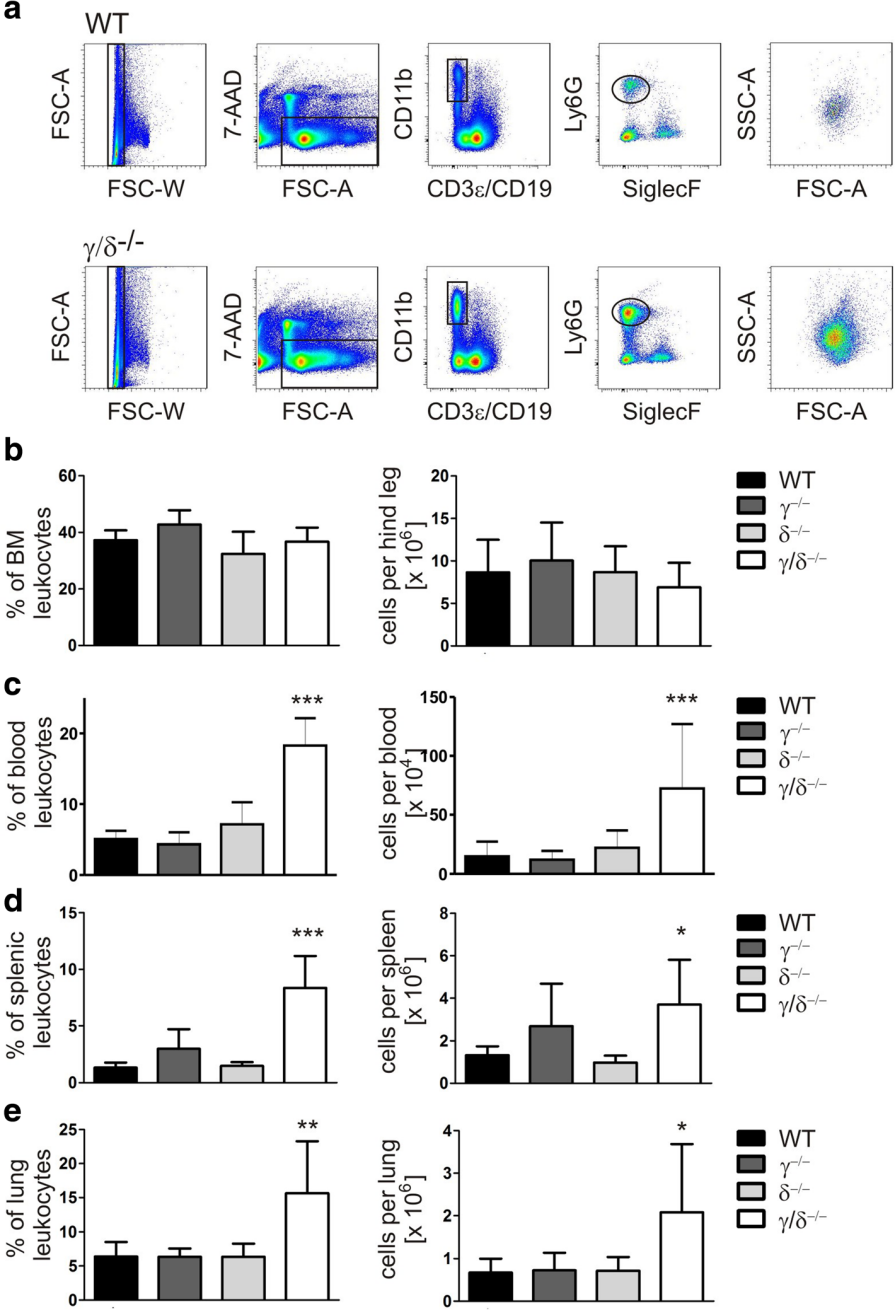
Increased neutrophil numbers in blood, spleen, and lung of p110γ/δ^−/−^ mice. To determine neutrophil numbers in WT, p110γ^−/−^, p110δ^−/−^, and p110γ/δ^−/−^ mice leukocyte suspensions from BM, blood, spleen, and lung were labeled with fluorescent antibodies and analyzed by flow cytometry. **a** Neutrophils were gated as singlet, live CD3ε^−^ CD19^−^ CD11b^+^ Siglec-F^−^ cells expressing Ly6G. Shown are representative examples of the gating strategy used for the analysis of neutrophils in spleen cell suspensions from one WT (top) and one p110γ/δ^−/−^ mouse (bottom). Back-gating of gated neutrophils on FSC-A / SSC-A plots (far-right, top and bottom) demonstrate the increased abundance of neutrophils in the p110γ/δ^−/−^ mouse (far-right bottom) when compared with the WT mouse (far-right, top). (**b - e**) Percentages and total numbers of gated neutrophils in (**b**) BM, (**c**) blood, (**d**) spleen, and (**e**) lung of WT, p110γ^−/−^, p110δ^−/−^, and p110γ/δ^−/−^ mice. Bars represent means + SD of *n* = 5–8 (BM, spleen), *n* = 12 (blood) or *n* = 4–11 (lung) mice per group. Data were analyzed by One-way ANOVA followed by Bonferroni’s comparison tests for selected pairs of columns. ***P < 0.001, **P < 0.01, *P < 0.05. Asterisks indicate differences in comparison to WT mice

Studies using p110γ/δ double knock-out mice showed that p110γ and p110δ are in concert required for the regulation of B- and T-cell homeostasis in mice [22, 28, 29]. We have previously observed that p110γ/δ^−/−^ mice exhibit an increased occurrence of neutrophils in the spleen [22]. Therefore, here we analyzed the potential mechanisms leading to this phenotype and investigated the role of p110γ and p110δ in neutrophil homeostasis. To compare neutrophil numbers in WT, p110γ^−/−^, p110δ^−/−^, and p110γ/δ^−/−^ mice leukocyte suspensions from BM, blood, spleen, and lung were stained with fluorescent antibodies against CD3ε, CD19, CD11b, Siglec-F, and Ly6G and measured by flow cytometry. Neutrophils were gated as singlet, live CD3ε^−^ CD19^−^ CD11b^+^ Siglec-F^−^ cells expressing the murine neutrophil defining antigen Ly6G (Fig. 1a). We found that frequencies and total numbers of neutrophils in the BM of p110γ^−/−^, p110δ^−/−^, and p110γ/δ^−/−^ mice were similar to WT mice (Fig. 1b). Analyses of blood, spleen, and lung revealed no significant differences in neutrophil percentages and total numbers between single deficient p110γ^−/−^ or p110δ^−/−^ mice and WT mice (Fig. 1c - e), although numbers of splenic neutrophils in p110γ^−/−^ mice tended to be increased compared to the WT group (Fig. 1d). In striking contrast, neutrophil percentages and numbers of p110γ/δ^−/−^ mice significantly exceeded those of WT mice in spleen, blood, and lung (Fig. 1a; c - e).

### p110γ/δ deficiency alters cell death rate of splenic neutrophils and granulocytic differentiation stages in the BM

**Fig. 2:**
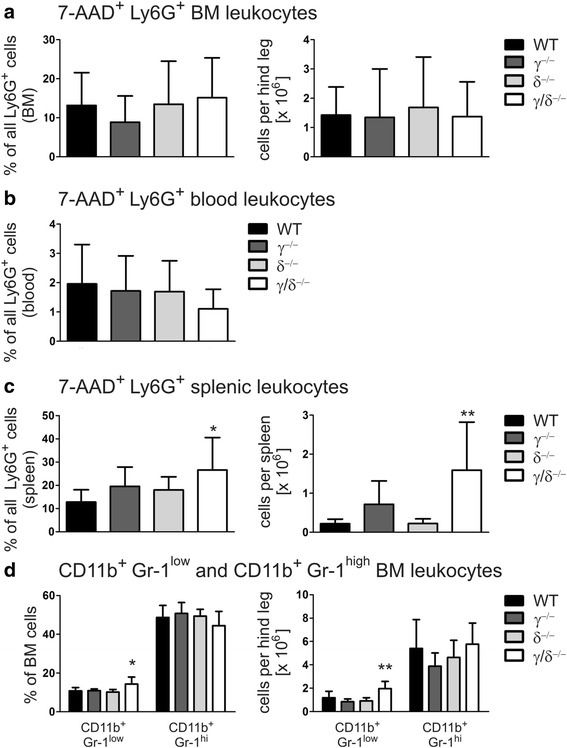
Increased neutrophil cell death and elevated CD11b^+^ Gr-1^low^ immature granulocytes in p110γ/δ^−/−^ mice. (**a-c**) Cell death of neutrophils was assessed by flow cytometry as measured by an increase in cell permeability of CD3ε^−^CD19^−^ CD11b^+^ Siglec-F^−^ Ly6G^+^ gated neutrophils to 7-AAD. Percentages of 7-AAD^+^ cells of gated neutrophils and total numbers of 7-AAD^+^ neutrophils in (**a**) BM, (**b**) blood, and (**c**) spleen of WT, p110γ^−/−^, p110δ^−/−^, and p110γ/δ^−/−^ mice. (**d**) Differentiating granulocytes in the BM were classified based on their expression of the cell surface markers CD11b and Gr-1. **d** Percentages (left panel) and total numbers (right panel) of CD11b^+^Gr-1^low^ and CD11b^+^Gr-1^high^ cells in WT, p110γ^−/−^, p110δ^−/−^, and p110γ/δ^−/−^ mice. Bars represent means + SD of *n* = 7–8 mice per group. Data were statistically analyzed as described in Fig. 1

To analyze whether neutrophilia in p110γ/δ^−/−^ mice resulted from an altered cell death rate we determined the number of 7-AAD^+^ dead neutrophils in BM, blood, and spleen. Numbers of 7-AAD^+^ neutrophils were comparable in BM and blood of WT, p110γ^−/−^, p110δ^−/−^, and p110γ/δ^−/−^ mice (Fig. 2a and b). There were also no significant differences in the numbers of 7-AAD^+^ splenic neutrophils between p110γ^−/−^ or p110δ^−/−^ and WT mice (Fig. 2c). However, in the spleen, frequencies and total numbers of 7-AAD^+^ neutrophils were significantly increased in p110γ/δ^−/−^ mice (Fig. 2c). These findings make it overall unlikely that neutrophilia in p110γ/δ^−/−^ mice was due to an increased neutrophil survival in the periphery.

Although numbers of mature neutrophils in the BM were not elevated in p110γ/δ^−/−^ mice, this did not rule out the possibility that neutrophilia in these animals resulted from an enhanced generation of early granulocytic maturation stages, followed by an enhanced release of mature granulocytes into the periphery. Differentiating granulocytes in the BM can be classified based on their expression of the cell surface markers CD11b and Gr-1; whereas immature granulocytes are CD11b^+^Gr-1^low^, mature granulocytes are CD11b^+^Gr-1^high^ [30]. The analysis revealed no differences in the frequencies or total numbers of CD11b^+^Gr-1^low^ or CD11b^+^Gr-1^high^ populations between p110γ^−/−^, p110δ^−/−^, and WT BM cells (Fig. 2d). However, the immature CD11b^+^Gr-1^low^ populations, but not the mature CD11b^+^Gr-1^high^ populations, were significantly higher in p110γ/δ^−/−^ mice than in p110γ^−/−^, p110δ^−/−^, and WT animals (Fig. 2d). Thus, it is feasible that elevated neutrophil numbers in blood and spleen of p110γ/δ^−/−^ mice resulted from an increased neutrophil production and an enhanced release of mature neutrophils from the BM.

### Homing of adoptively transferred neutrophils is not significantly altered in p110γ/δ^−/−^ mice

Neutrophil homeostasis is also dependent on a chemokine-induced recruitment of these cells to different tissues and organs. Experiments using mice with a myeloid lineage-specific deletion of the chemokine receptor CXCR4 demonstrated that CXCR4 directs the homing of adoptively transferred neutrophils to the BM of unconditioned WT recipients [31]. It has been shown that CXCR4 is up-regulated on BM neutrophils as they age. Moreover, adoptive transfer experiments suggested that in vitro-aged senescent neutrophils home to the bone marrow in a CXCR4-dependent manner [26]. Interestingly, p110γ and class IA PI3Ks play a role in SDF-1α-induced CXCR4-dependent leukocyte migration [10, 32]. Therefore, we assumed that the abnormal accumulation of neutrophils in blood and spleen of p110γ/δ^−/−^ mice resulted from a defective CXCR4-dependent homing of (senescent) neutrophils to the BM. To test this, we analyzed the homing of adoptively transferred “young” and “senescent” neutrophils in WT and p110γ/δ^−/−^ mice using a refined method described by Martin et al. [26] (for details see Materials and Methods). When comparing retrieved neutrophils within each genotype, we found that the frequencies of “young” 0 h and of “aged” 6 h neutrophils were not significantly different in blood and spleen (Additional file 1: Figure S1). Surprisingly, similar results were obtained in the BM. This outcome is in conflict with findings of others who observed that following co-injection of Ly6G (Gr1)-APC-labeled “aged” neutrophils and Ly6G (Gr1)-FITC-labeled “young” neutrophils, the APC-labeled “aged” neutrophil population dominated in the BM of the recipients [26]. We have previously shown that coupled fluorochromes can alter the functional properties of neutrophil-targeting Ly6G antibodies [25] and assume that the conflicting results might be explained by the cell-depleting effect of the FITC-coupled Ly6G antibody.

Next, we compared the retrieval rates of 0 h neutrophils or 6 h neutrophils between WT and p110γ/δ^−/−^ mice. Interestingly, there were no significant differences detected between WT and p110γ/δ^−/−^ retrieval rates of 0 h neutrophils in each organ (Additional file 1: Figure S1). Similarly, the analysis of the retrieval rates of 6 h neutrophils showed no significant differences between WT and p110γ/δ^−/−^ mice (Additional file 1: Figure S1). In conclusion, these findings do not support the hypothesis that a defective BM-homing plays a major role in increasing peripheral neutrophil levels in p110γ/δ^−/−^ mice.

### p110γ/δ deficiency increases serum concentrations of G-CSF and neutrophil mobilizing chemokines

**Fig. 3:**
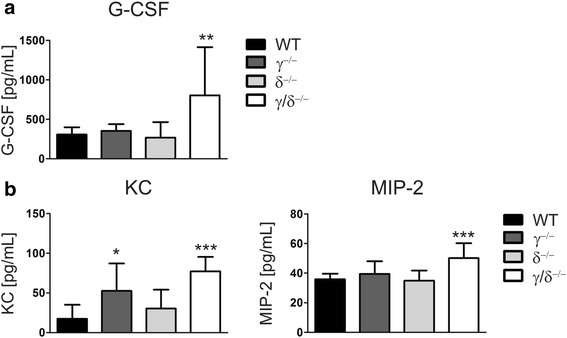
Serum concentrations of G-CSF and CXC chemokines are increased in p110γ/δ^−/−^ mice. Cytokines were measured using a Bio-Plex assay. Concentrations of (**a**) G-CSF and (**b**) the CXC chemokines KC and MIP-2 in the serum of WT, p110γ^−/−^, p110δ^−/−^, and p110γ/δ^−/−^ mice. Bars represent means + SD of *n* = 10 mice per group. Data were statistically analyzed as described in Fig. 1

Generation, release, and survival of neutrophils are mediated by various cytokines, chemokines and growth factors and the interaction with their respective receptors. To determine if these mediators were altered in p110γ^−/−^, p110δ^−/−^, and p110γ/δ^−/−^ mice, various cytokines were measured simultaneously using a Bio-Plex assay (Table 1). G-CSF is the major growth factor for neutrophils and it regulates their proliferation and differentiation [3, 4]. Importantly, while serum concentrations of G-CSF were similar between p110γ^−/−^, p110δ^−/−^, and WT mice, they were significantly up-regulated more than two-fold in p110γ/δ^−/−^ animals (Fig. 3a). Consistent with this, G-CSF concentrations in the serum significantly correlated with total neutrophil numbers in the spleen (Spearman rank correlation: *r* = 0.68; *P* < 0.01).

In addition to its effect on neutrophil production, G-CSF may also stimulate the release of neutrophils from the BM by down-regulating CXCL12/SDF-1α and by reducing CXCR4 expression on neutrophils in the BM [31]. However, analysis of CXCL12/SDF-1α concentrations in the BM as well as measurement of CXCR4 expression on neutrophils revealed no significant difference between mice of all four genotypes (Additional file 2: Figure S2a and b). The release of neutrophils is also mediated by the CXC chemokines CXCL1/KC and CXCL2/MIP-2. Interaction of CXCL1/KC and CXCL2/MIP-2 with their receptor CXCR2 acts to mobilize neutrophils from the BM into the circulation [2]. CXCR2 expression on neutrophils showed no significant difference between all four genotypes (Additional file 2: Figure S2c). Interestingly, however, in comparison to WT mice, serum concentrations of both KC and MIP-2 were significantly elevated in p110γ/δ^−/−^ mice (Fig. 3b). Additionally, KC was also elevated in p110γ^−/−^ mice (Fig. 3b). This demonstrates that deficiency of p110γ/δ in p110γ/δ^−/−^ mice results in increased serum concentrations of major neutrophilic growth and release factors.

### p110γ/δ deficiency elevates IL-17A levels and increases the number of IL-17^+^ T cells

**Fig. 4:**
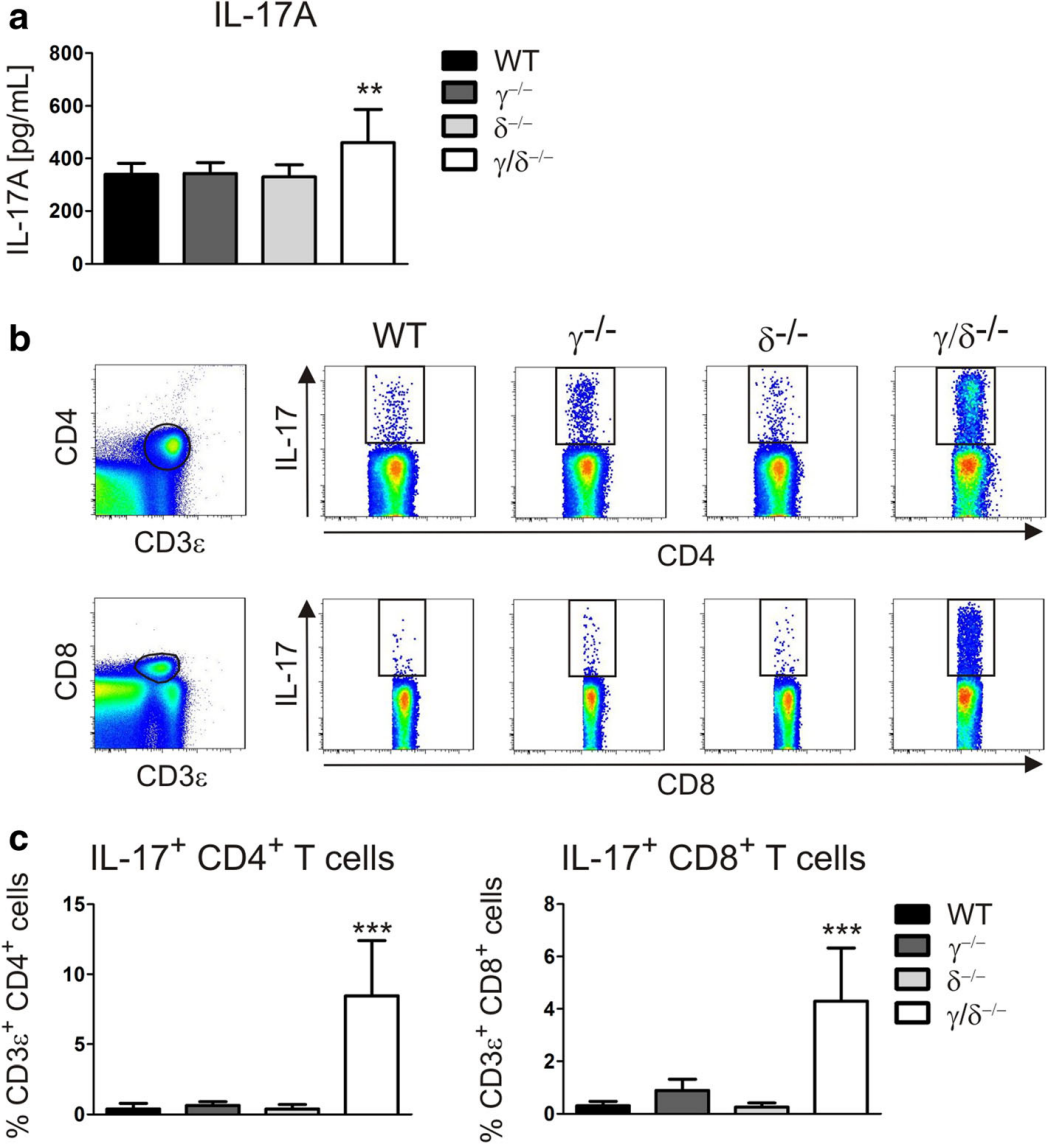
Elevated IL-17A levels and increased numbers of IL-17-producing T cells in p110γ/δ^−/−^ mice. IL-17A serum concentrations were measured using a Bio-Plex assay, and IL-17A-producing T cell subsets in the spleen were analyzed by flow cytometry. **a** Serum concentration of IL-17A in WT, p110γ^−/−^, p110δ^−/−^, and p110γ/δ^−/−^ mice (*n* = 10). Data were statistically analyzed as described in Fig. 1. **b** CD3ε^+^CD4^+^ (far-left, top) and CD3ε^+^CD8^+^ T cells (far-left, bottom) were gated from pre-gated singlet live NK1.1^−^ splenic leukocytes and subsequently analyzed for IL-17A expression. Shown are representative examples of the analysis of IL-17A expression in CD3ε^+^CD4^+^ and CD3ε^+^CD8^+^ T cell subsets from WT, p110γ^−/−^, p110δ^−/−^, and p110γ/δ^−/−^ mice. **c** Percentages of IL-17A^+^ subpopulations of CD3ε^+^ CD4^+^ or CD3ε^+^ CD8^+^ T cell subsets in WT, p110γ^−/−^, p110δ^−/−^, and p110γ/δ^−/−^ mice (*n* = 4–10). Data were statistically analyzed as described in Fig. 1

The production of G-CSF and the neutrophil mobilizing chemokines CXCL1/KC and CXCL2/MIP-2 can be induced by IL-17 [33–35]. While the analysis of serum concentrations of IL-17A revealed no differences between WT, p110γ^−/−^, and p110δ^−/−^ mice, IL-17A levels were significantly elevated in p110γ/δ^−/−^ mice (Fig. 4a). We also found that IL-17A concentrations in the serum positively correlated with G-CSF levels (Spearman rank correlation: *r* = 0.45; *P* < 0.01).

Important sources of IL-17A are IL-17-producing T cell subsets [6]. In line with the above findings, flow cytometric measurement of intracellular IL-17A expression in pre-gated CD4^+^ or CD8^+^ T cells (Fig. 4b) demonstrated that the frequencies of IL-17-producing T cells were drastically increased in p110γ/δ^−/−^ mice (Fig. 4c).

### p110γ/δ deficiency results in a percentage increase in cytokine producing T cell subsets

**Fig. 5:**
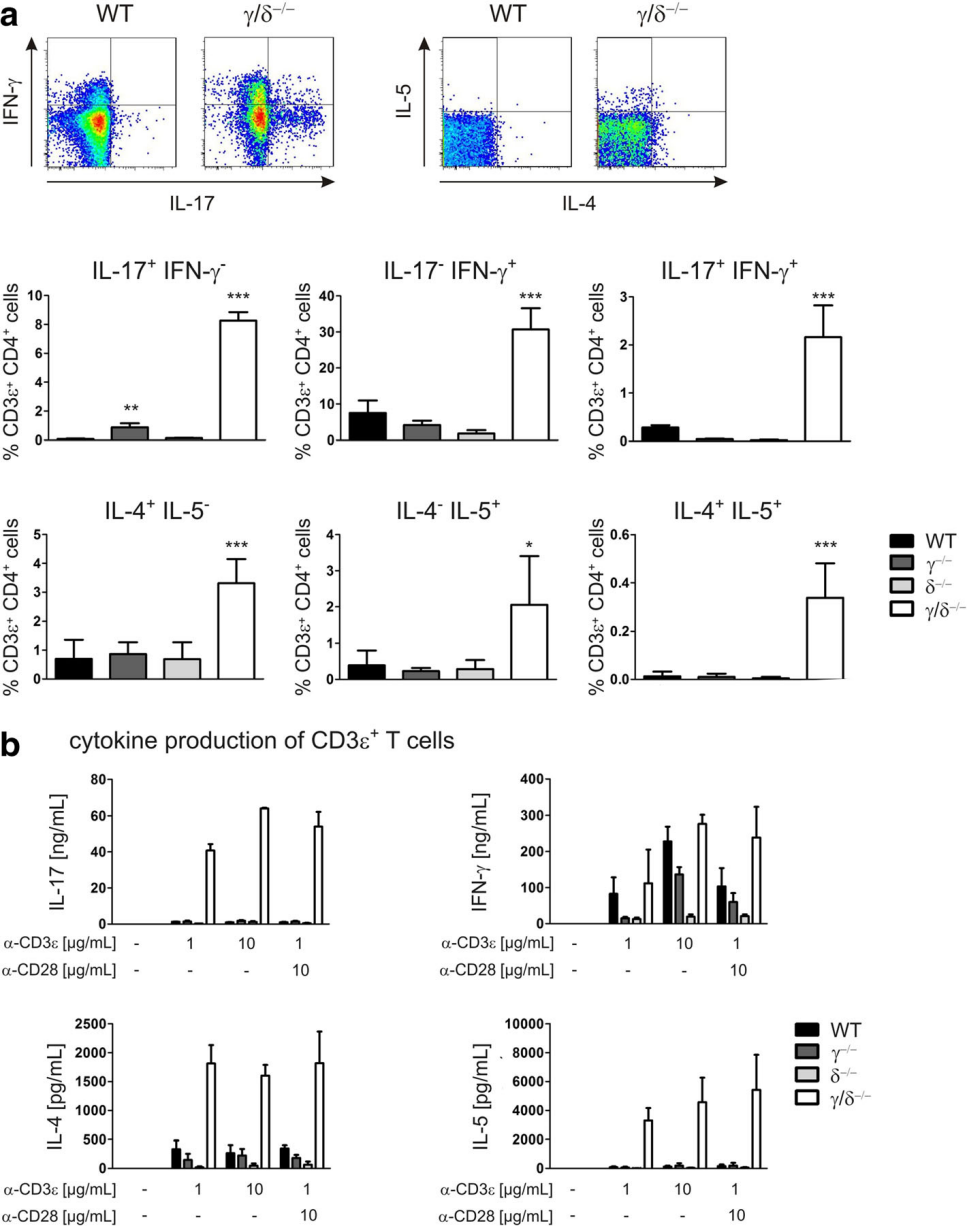
Frequencies of cytokine producing T cell subsets are increased in p110γ/δ^−/−^ mice. To determine frequencies of cytokine producing T helper cell (Th cell) subsets, PMA-stimulated splenocytes were measured by flow cytometry. Th cells were gated as singlet live NK1.1^−^ CD3ε^+^ CD4^+^ cells and analyzed for intracellular IL-17A, IFN-γ, IL-4, and IL-5 expression. Cytokine secretion by MACs-purified splenic T cells was determined following incubation with indicated concentrations of plate-coated anti-CD3ε or anti-CD3ε/anti-CD28 and IL-17A, IFN-γ, IL-4, and IL-5 concentrations in the supernatants were measured by ELISA. **a** Cytokine producing Th cell subsets in the spleens of WT, p110γ^−/−^, p110δ^−/−^, and p110γ/δ^−/−^ mice. Flow cytometry plots show representative examples of the analysis of IL-17, IFN-γ, IL-4, and IL-5 expression in CD3ε^+^ CD4^+^ T cells from one WT and one p110γ/δ^−/−^ mouse and graphs display percentages of cytokine producing Th cell subsets. Bars represent means + SD of pooled data from three independent experiments. **b** Production of IL-17A, IFN-γ, IL-4, and IL-5 by T cells from WT, p110γ^−/−^, p110δ^−/−^, and p110γ/δ^−/−^ mice. Bars represent means + SD of pooled data from three independent experiments carried out in duplicates

Mice lacking p110γ and expressing a catalytically inactive p110δ isoform (p110γ^−/−^δ^C910A^ mice) show a skewing toward Th2 immune responses [36]. Interestingly, our data demonstrated that, in addition to IL-17A, serum levels of the Th2 cytokine IL-5 were also significantly increased in p110γ/δ^−/−^ mice compared to WT mice (Table 1; [24]). Thus, to investigate whether deficiency in p110γ/δ also resulted in a relative increase in other cytokine-producing T cell subsets, PMA-stimulated spleen cells were studied by flow cytometry. T helper cells were gated as 7-AAD^−^ CD3ε^+^ CD4^+^ NK1.1^−^ singlet leukocytes and were analyzed for the intracellular expression of the cytokines IL-17A, IFN-γ, IL-4 and IL-5. As exemplarily shown in Fig. 5a, not only the frequency of IL-17^+^ IFN-γ^−^, but also of IL-17^−^ IFN-γ^+^, IL-17^+^ IFN-γ^+^, IL-4^+^ IL-5^−^, IL-4^−^ IL-5^+^, and IL-4^+^ IL-5^+^ CD4^+^ T cell subsets were increased in p110γ/δ^−/−^ mice compared to WT mice. Moreover, MACS-purified CD3ε- or CD3ε/CD28-stimulated T cells from p110γ/δ^−/−^ mice, but not from p110γ^−/−^ or p110δ^−/−^ mice, secreted more IFN-γ and produced drastically elevated amounts of IL-17A, IL-4, and IL-5 than T cells from WT mice (Fig. 5b). This clearly indicates that p110γ/δ deficiency in mice causes a general percentage increase in cytokine producing T cell subsets, including IL-17^+^ T cells.

### IL-17 stimulation of p110γ/δ^−/−^ lung tissue cells induces normal Akt phosphorylation and G-CSF and KC production

Collectively, these results are in line with the hypothesis that p110γ/δ deficiency in mice causes a relative increase in IL-17 cells and systemic levels of IL-17, which then lead to up-regulation of G-CSF, neutrophil releasing chemokines, and neutrophilia.

**Fig. 6:**
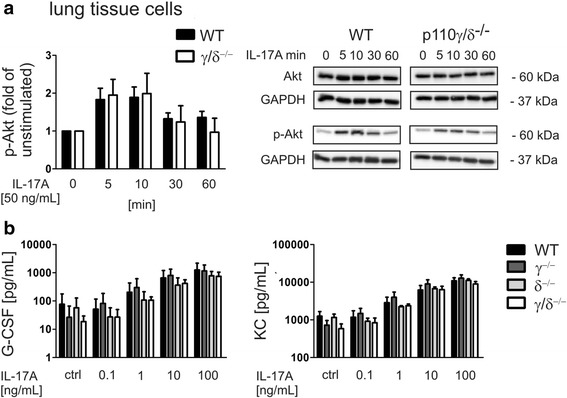
IL-17A stimulation of lung tissue cells from p110γ/δ^−/−^ mice induces normal Akt phosphorylation and G-CSF and KC production. To measure IL-17-induced Akt phosphorylation cultivated tissue cells from lungs of WT and p110γ/δ^−/−^ mice were stimulated with 50 ng/mL IL-17A. Cells were harvested at the indicated time points upon which whole cell lysates were subjected to immunoblot analysis using anti-phospho-Akt, anti-Akt, and anti-GAPDH antibodies. IL-17A induced cytokine production of lung tissue cells from WT, p110γ^−/−^, p110δ^−/−^, and p110γ/δ^−/−^ mice was measured by ELISA. **a** Phospho-Akt expression in lung tissue cells from WT and p110γ/δ^−/−^ mice at different time points following IL-17A stimulation. Depicted is a statistical evaluation of phospho-Akt levels and representative blots showing the expression of phospho-Akt, total Akt, and GAPDH. Bars present the average fold change + SD of phospho-Akt levels of unstimulated cells. Phospho-Akt and Akt were first normalized to GAPDH to control for protein loading differences and then phospho-Akt was normalized to Akt levels. Bars represent means + SD of pooled mean values from three independent experiments, each measured in triplicates or quadruplicates. **b** Dose-dependent production of G-CSF and KC by lung tissue cells from WT, p110γ^−/−^, p110δ^−/−^, and p110γ/δ^−/−^ mice that were stimulated with increasing concentrations of IL-17A for 24 h. Bars represent means + SD of pooled mean values from three independent experiments measured in triplicates

However, this interpretation appears to be in conflict with findings by Harris et al.*,* who demonstrated that PI3Kγ is signaling downstream of the IL-17A receptor and showed that IL-17-induced Akt phosphorylation and cytokine production in T cells require PI3Kγ [21]. IL-17 induces the production of G-CSF, CXCL1/KC, and CXCL2/MIP-2 in tissue cells like fibroblasts, epithelial and endothelial cells [33–35]. Thus, to test whether tissue cells from p110γ^−/−^, p110δ^−/−^, and p110γ/δ^−/−^ mice are capable of responding to IL-17 stimulation, we established a primary cell culture of lung tissue cells. Immunoblot analysis of p110α, p110β, p110γ, and p110δ proteins confirmed that all four p110 isoforms were expressed in WT lung tissue cells, whereas the p110γ and p110δ isoforms were lacking in p110γ/δ^−/−^ cells (Additional file 3: Figure S3a). We observed that IL-17A stimulation resulted in a comparable Akt and p38 phosphorylation not only in WT cells but also in p110γ/δ^−/−^ cells (Fig. 6a and Additional file 3: Figure S3b, c). Additionally, we demonstrated that stimulation with increasing concentrations of IL-17A induced a dose-dependent production of G-CSF and KC, in lung tissue cells not only from WT mice, but also from p110γ^−/−^, p110δ^−/−^, and p110γ/δ^−/−^ mice (Fig. 6b). While these results appear to confirm a role for PI3Ks in IL-17 signaling pathways, they on the other hand also demonstrate that the p110γ and p110δ isoforms are not critically required for the IL-17 induced Akt or p38 phosphorylation and production of G-CSF and KC.

Taken together, this suggests that not only in WT, but also in p110γ^−/−^, p110δ^−/−^, and p110γ/δ^−/−^ mice IL-17 can induce a dose-dependent production of G-CSF and KC.

## Discussion

The predominantly hematopoietically expressed PI3K catalytic isoforms p110γ and p110δ have numerous important roles in immune regulation. Studies using p110γ/δ double knock-out mice showed that the two isoforms work in concert to regulate B and T cell homeostasis [22, 29, 36]. Here we expand the function of p110γ and p110δ and demonstrate a crucial role for both PI3K isoforms in neutrophil homeostasis.

Two major factors controlling neutrophil homeostasis are the generation of these cells in the BM and their cell death rate in the periphery. Studies analyzing neutrophils in p110γ and p110δ single knock-out mice suggest that p110γ might specifically play a role in regulating neutrophil homeostasis. Whereas p110δ^−/−^ mice showed normal neutrophil numbers [23], p110γ^−/−^ mice were reported to have increased neutrophil counts in the blood but not in the BM [10, 13]. Moreover, p110γ^−/−^ mice exhibited an enhanced neutrophil apoptosis [37]. Our findings that the total number and the cell death rate of splenic neutrophils were strikingly increased in p110γ/δ double knock-out mice emphasize a requirement for both isoforms in controlling the homeostasis of neutrophils.

The increased cell death of splenic neutrophils in p110γ/δ^−/−^ mice did not explain the occurrence of neutrophilia in these animals. Nevertheless, increased numbers of early granulocytic differentiation stages in the BM and elevated serum levels of G-CSF might suggest that neutrophilia in p110γ/δ^−/−^ mice resulted from an increased production and release of neutrophils from the BM. G-CSF is the principal growth factor directly regulating the proliferation and differentiation of neutrophils [3, 4], but it also modifies neutrophil survival by delaying apoptosis [38]. Upon binding to its receptor G-CSF stimulates several signaling cascades, including the Jak/Stat, Ras-Raf-MAP kinase, and Src family pathway (references in [39]). The G-CSF-dependent regulation of granulopoiesis occurs via Jak/Stat signaling pathways (references in [40]]. These pathways are functionally separated from the G-CSF-induced stimulation of the Src family pathway, which leads to PI3K activation followed by Akt phosphorylation [39]. PI3K activation is required for the G-CSF-induced delay of spontaneous neutrophil apoptosis [41]. Thus, functionally separated signaling pathways might explain why elevated G-CSF levels in p110γ/δ^−/−^ mice correlated with increased neutrophil numbers, although at the same time the cell death rate of neutrophils was elevated in these animals.

G-CSF not only drives granulopoiesis and delays neutrophil apoptosis, but it additionally decreases CXCL12/SDF-1α levels and reduces CXCR4 expression on neutrophils in the BM [31]. Mature neutrophils are retained in the BM by the CXCL12/SDF-1α-CXCR4 axis [31]. Therefore, the down-regulation of CXCL12/SDF-1α and CXCR4 acts to facilitate the release of neutrophils from the BM. Neutrophil mobilization from the BM is also induced by the CXC chemokines CXCL1/KC and CXCL2/MIP-2 [2]. Whereas our data revealed no significant effect of p110γ/δ deficiency on CXCL12/SDF-1α, CXCR4 or CXCR2 expression, increased serum concentrations of CXCL1/KC and CXCL2/MIP-2 may contribute to neutrophilia in p110γ/δ^−/−^ mice. However, as neutrophil emigration in response to CXCL1/KC and CXCL2/MIP-2 is dependent on the activity of PI3Kγ and/or PI3Kδ [15], it is unclear, whether normal CXCL1/KC and CXCL2/MIP-2 mediated mobilization of neutrophils occurs in p110γ/δ^−/−^ mice. On the other hand, CXCL12/SDF-1α signaling involves p110γ and class I PI3Ks [10, 32]. Thus, an increased release of mature neutrophils (and therefore increased numbers of mature neutrophils in the periphery and normal numbers in the BM) may also be caused by an impaired CXCL12/SDF-1α-CXCR4-mediated retention of mature neutrophils in the BM of p110γ/δ^−/−^ mice.

To test this hypothesis, we have attempted the analysis of the motility of BM-derived neutrophils in response to CXCL12/SDF-1α using a transwell assay. Neutrophils from p110γ/δ^−/−^ mice showed a reduced response compared to WT cells (Additional file 4: Figure S4). However, as our results were strongly time- and concentration-dependent, it is currently unclear in how far such in vitro assays reflect the in vivo situation.

Importantly, CXCL12/SDF-1α-CXCR4-mediated signaling also regulates the homing of neutrophils from the blood to the BM [31], and adoptive transfer experiments using in vitro-aged BM cells were interpreted to show that senescent neutrophils are recruited to the bone marrow in a CXCR4-dependent manner [26]. This suggested the possibility that neutrophilia in p110γ/δ^−/−^ mice resulted from a defective CXCL12/SDF-1α-CXCR4-mediated homing of (senescent) neutrophils to the BM. To test this hypothesis, we analyzed the retrieval rates of adoptively co-transferred “young” and “senescent” neutrophils in blood, spleen, and BM of WT and p110γ/δ^−/−^ mice. However, as neither the homing of “young” nor of “aged” neutrophils was significantly different between the WT and the p110γ/δ^−/−^ group, it appears overall unlikely that a defective neutrophil homing plays a major role in increasing peripheral neutrophil numbers in p110γ/δ^−/−^ mice.

It was described that over-expression of IL-17 in mice results in neutrophilia [5]. This suggested that in p110γ/δ^−/−^ mice increased systemic concentrations of G-CSF were driven by increased IL-17 levels. However, this interpretation appears to be in conflict with previous work of Harris demonstrating that PI3Kγ integrates in IL-17 signaling pathways [21]. In this study the authors showed that IL-17-induced cytokine production and Akt and NF-κB phosphorylation were absent in in vitro-stimulated PI3Kγ^−/−^ T cells, and demonstrated that following intranasal installation of IL-17 Akt signaling was strongly attenuated in the lung tissue of PI3Kγ^−/−^ mice [21]. In contrast, our analyses of in vitro-cultured primary lung tissue cells confirmed that IL-17 was indeed capable of inducing Akt and p38 phosphorylation not only in WT but also in p110γ/δ^−/−^ cells and demonstrated that IL-17 could stimulate a dose-dependent G-CSF and CXCL1/KC production in WT as well as in single knock-out and p110γ/δ^−/−^ cells. The discrepancies in the role of PI3Kγ in IL-17 signaling between our work and the study of Harris et al. might relate to cell type dependent differences in IL-17A signaling and/or to major differences in the experimental setting [21]. Whereas Harris et al. investigated IL-17A-induced Akt phosphorylation and cytokine production following in vitro-stimulation of splenic T cells and following intranasal installation of IL-17A, in our study IL-17A-induced Akt and p38 phosphorylation and G-CSF and KC production was analyzed in primary lung tissue cells.

Interestingly, p110α and p110β were both expressed in lung tissue cells from WT and p110γ/δ^−/−^mice, which might also explain the lack of effect of p110γ/δ deficiency on IL-17A-induced Akt and p38 phosphorylation, G-CSF and CXC chemokine production based on a functional compensation by p110α and/or p110β.

Studies investigating the role of p110γ or p110δ in IL-17 responses demonstrated that both isoforms are involved in Th17 cell differentiation and IL-17 production [16–18]. Moreover, loss of class IA PI3K regulatory subunit p85 [20] or pharmacological inhibition of p110γ [19] or p110δ [20] reduced anti-CD3ε/IL-6/TGF-β-driven Th17 cell differentiation and IL-17 production, whereas the IL-12- or IL-4-primed differentiation of Th1 or Th2 cells was unaffected [20]. In striking contrast, our data demonstrated that, although total CD3ε T cell numbers of p110γ/δ mice were reduced by almost 10-fold in comparison to WT mice [22, 28], dual deficiency in p110γ and p110δ significantly increased frequencies of CD4^+^ and CD8^+^ IL-17 producing T cell subsets and IL-17 levels in vivo. Interestingly, anti-CD3ε/IL-6/TGF-β-driven Th17 cell differentiation and IL-17A production are also unimpaired in naïve CD4^+^ splenocytes from PI3Kγ^KD/KD^ mice [19], which collectively suggests that following genetic inactivation of the catalytic activity of p110γ and p110γ/δ compensatory mechanisms via other functionally redundant PI3K isoforms might occur.

Elevated IL-17^+^ T cell numbers were accompanied by increased frequencies of IL-4^+^ and IL-5^+^ T cell subsets and higher serum levels of IL-5, suggesting that IL-17^+^ T cells were up-regulated in the context of a general percentage increase of other cytokine producing T cell subsets. The reason why frequencies of cytokine-producing T cell subsets were increased in p110γ/δ^−/−^ mice is unclear. However, mice lacking the catalytical activities of p110γ/δ suffer from lymphopenia and their T cells exhibit an activated phenotype [28, 36]. Interestingly, lymphopenia in mice results in homeostatic expansion of remaining T cells, which acquire an effector/memory phenotype [42]. Therefore, it has been suggested that increased T cell cytokine responses in p110γ^−/−^δ^C910A^ mice are secondary to lymphopenia and result from homeostatic expansion of the few T cell clones that developed [36].

While in p110γ/δ^−/−^ mice percentages of splenic IL-4^+^ and IL-5^+^ T cell subsets were increased to a similar degree as IFN-γ^+^ T cell subsets, percentages of IL-17^+^ IFN-γ^−^ T cells were disproportionally elevated by more than 100-fold. These cells were also significantly increased in p110γ^−/−^ mice, albeit to a much lower extent. Interestingly, increases in IL-17^+^ T cells in p110γ^−/−^ and p110γ/δ^−/−^ mice corresponded with an elevated IL-23 mRNA expression in splenic APCs in both groups (data not shown). IL-23 is a growth and stabilization factor for IL-17-producing Th17 cells [6]. Thus, it is possible that the disproportional expansion of IL-17^+^ IFN-γ^−^ T cells in p110γ/δ^−/−^ mice was additionally promoted by an elevated IL-23 production.

## Conclusions

In summary, p110γ/δ deficiency in mice resulted in complex immunological changes, which might in concert contribute to neutrophilia. Our data suggest that increased neutrophil numbers in p110γ/δ^−/−^ mice are accompanied by a disrupted control of the generation of cytokine-producing T cell subsets, up-regulation of IL-17^+^ T cells, and increased IL-17/G-CSF signaling. In addition to neutrophilia, p110γ/δ^−/−^ mice have been demonstrated to suffer from B- and T-cell lymphopenia [22, 28, 29] and eosinophilia [24]. This shows that dual genetic inactivation of p110γ/δ leads to an immunological phenotype which cannot be predicted by the short-term use of single inhibitors, single deficient mice, or the short-term application of dual inhibitors in in vitro experiments or disease models. It is unclear, whether predictions can be made from the double knock-out model to the effect of long-term treatment with dual p110γ/δ inhibitors. Nevertheless, it has been shown that similar to genetic ablation, pharmacological inactivation of p110γ/δ with the dual-specific inhibitor CAL-130 results in a profound depletion of thymocytes in WT mice [43], suggesting that long-term treatment induces lymphopenia. If such a lymphopenia is indeed regulating T cell activation and driving the expansion of cytokine-producing T cell subsets, then these findings may have serious implications for the treatment with dual pharmacological inhibitors of p110γ and p110δ. Moreover, in clinical studies using the single p110δ-specific inhibitor Idelalisib, severe infections and infection-related fatal outcomes occurred [44] and warn against even more severe outcomes during long-term treatment with dual p110γ/δ inhibitors.

## Additional files

Additional file 1: Figure S1. Homing of adoptively transferred neutrophils is similar in p110γ/δ^−/−^ mice and WT mice. Equal fractions of “senescent” neutrophils, contained in BM cell suspensions that had been incubated for 6 h in vitro (6 h neutrophils), and “young” neutrophils, contained in freshly isolated BM (0 h neutrophils), were stained with Ly6G (1A8)-APC (6 h neutrophils) or Ly6G (1A8)-PE (0 h neutrophils), and were co-injected into recipient mice. WT 0 h and 6 h BM cells were injected into WT mice, whereas p110γ/δ^−/−^ 0 h and 6 h BM cells were injected into p110γ/δ^−/−^ mice, respectively. One hour later, leukocytes from recipients were stained with antibodies against CD3ε, CD11b, and CD19 and were analyzed by flow cytometry. Myeloid cells were gated as CD11b^+^ CD3ε^−^ CD19^−^ singlet leukocytes. They were analyzed for the presence of 1A8-APC- or –PE-labeled neutrophils. Graphs show Ly6G (1A8)-PE-labeled 0 h neutrophils and Ly6G (1A8)-APC-labeled 6 h neutrophils retrieved in BM, blood, and spleen of recipient mice. Retrieved cells are expressed as percentages of injected Ly6G (1A8)-labeled cells. Bars represent means + SD of *n* = 5 mice per group. (JPEG 73 kb)

Additional file 2: Figure S2. Expression of CXCR4 and CXCL12/SFD-1α in WT, p110γ^−/−^, p110δ^−/−^, and p110γ/δ^−/−^ mice. **a** To measure CXCL12/SDF-1α concentrations in the BM, tibias were flushed with 500 μl PBS, cells were pelleted and supernatants were subsequently subjected to ELISA. Bars represent means + SD of *n* = 9–10 mice per group. **b** To determine CXCR4 expression leukocyte suspensions were labeled with fluorescent antibodies and analyzed by flow cytometry. Neutrophils were gated as singlet, live CD3**ε** ^−^ CD19^−^ CD11b^+^ Siglec-F^−^ Ly6G^+^ cells and were analyzed for the expression of CXCR4 (CD184). Shown are GMFI of CD184-APC of gated neutrophils in BM (left), blood (middle) and spleen (right). Bars represent means + SD of *n* = 5–8 mice per group. **c** To determine CXCR2 expression leukocyte suspensions were labeled with fluorescent antibodies and analyzed by flow cytometry. Neutrophils were gated as singlet, live CD3**ε** ^−^ CD19^−^ CD11b^+^ Siglec-F^−^ Ly6G^+^ cells and were analyzed for the expression of CXCR2 (CD182). Shown are GMFI of CD182-APC of gated neutrophils in BM (left), blood (middle) and spleen (right). Bars represent means + SD of *n* = 7–8 mice per group. (JPEG 248 kb)

Additional file 3: Figure S3. Protein expression of p110α, p110β, p110γ, and p110δ isoforms in primary lung tissue cells from WT and p110γ/δ^−/−^ mice. **a** Analysis of p110 protein expression in cultured tissue cells from lungs of WT and p110γ/δ^−/−^ mice was performed by immunoblot analysis using anti-p110α, anti-p110β, anti-p110γ, and anti-p110δ specific antibodies. Re-probing for GAPDH served to confirm equal protein loading. **b** Phospho-p38 expression in lung tissue cells from WT and p110γ/δ^−/−^ mice at different time points following IL-17A (50 ng/ml) stimulation. The blot shows the expression of phospho-p38 and total p38. Re-probing for tubulin served to confirm equal protein loading. **c** Depicted is a statistical evaluation of phospho-p38 levels. Bars present the average fold change + SD of phospho-p38 levels of unstimulated cells. Phospho-p38 and p38 were first normalized to Tubulin to control for protein loading differences and then phospho-p38 was normalized to p38 levels. Bars represent means + SD of pooled mean values from three independent experiments**.** (JPEG 151 kb)

Additional file 4: Figure S4. Migration of neutrophils from WT and p110^−/−^ mice upon CXCL12/SDF-1. Transwells inserts (3.0 μm pore size, Costar) were placed in 24-well plates containing CXCL12/SDF-1(10 nM or 50 nM). BM cells labeled with 40 Ly6G-APC were seeded on the upper chamber of each well at 3 × 106 cells per well. In parallel, cells were directly seeded into wells without transwell inserts and served as positive controls (100% migration). Cells were incubated for 60 or 110 min. Then Ly6G^+^ neutrophils in the lower chambers were counted by flow cytometry. The number of migrated neutrophils is expressed as % of positive control. Bars present means + SD from one experiment measured in duplicates. (JPEG 112 kb)

## Abbreviations

7-AAD: 7-aminoactinomycin D
APCs: antigen presenting cells
BM: bone marrow
DAPI: 4’,6-diamidino-2-phenylindole
Jak: Janus kinase
MACS: magnetic-activated cell sorting
PDK1: phosphoinositide dependent protein kinase-1
PMA: phorbol 12-myristate 13-acetate
Stat: signal transducer and activator of transcription
Th cell: T helper cell
