# Supplementary figures and images for: Deficiency of PI3-Kinase catalytic isoforms p110γ and p110δ in mice enhances the IL-17/G-CSF axis and induces neutrophilia
Source: Cell Commun Signal. 2017 Jul 19;15:28. doi: 10.1186/s12964-017-0185-y (PMC5518148; doi:10.1186/s12964-017-0185-y)

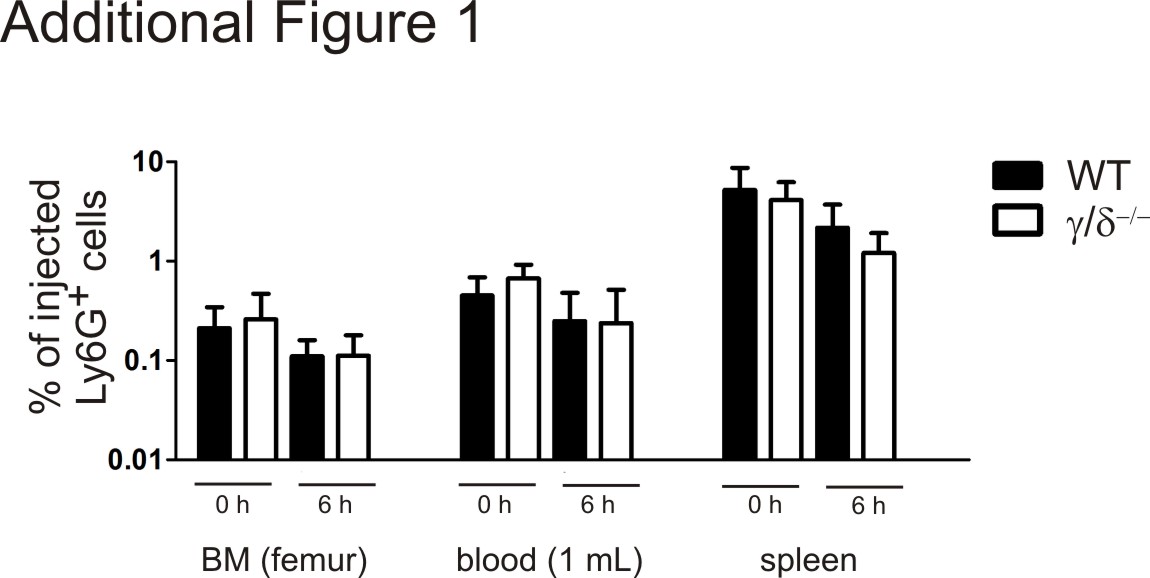

Supplement: Supplementary file 1 — Homing of adoptively transferred neutrophils is similar in p110γ/δ−/− mice and WT mice. Equal fractions of “senescent” neutrophils, contained in BM cell suspensions that had been incubated for 6 h in vitro (6 h neutrophils), and “young” neutrophils, contained in freshly isolated BM (0 h neutrophils), were stained with Ly6G (1A8)-APC (6 h neutrophils) or Ly6G (1A8)-PE (0 h neutrophils), and were co-injected into recipient mice. WT 0 h and 6 h BM cells were injected into WT mice, whereas p110γ/δ−/− 0 h and 6 h BM cells were injected into p110γ/δ−/− mice, respectively. One hour later, leukocytes from recipients were stained with antibodies against CD3ε, CD11b, and CD19 and were analyzed by flow cytometry. Myeloid cells were gated as CD11b+ CD3ε− CD19− singlet leukocytes. They were analyzed for the presence of 1A8-APC- or –PE-labeled neutrophils. Graphs show Ly6G (1A8)-PE-labeled 0 h neutrophils and Ly6G (1A8)-APC-labeled 6 h neutrophils retrieved in BM, blood, and spleen of recipient mice. Retrieved cells are expressed as percentages of injected Ly6G (1A8)-labeled cells. Bars represent means + SD of n = 5 mice per group. (JPEG 73 kb) [file 12964_2017_185_MOESM1_ESM.jpg]

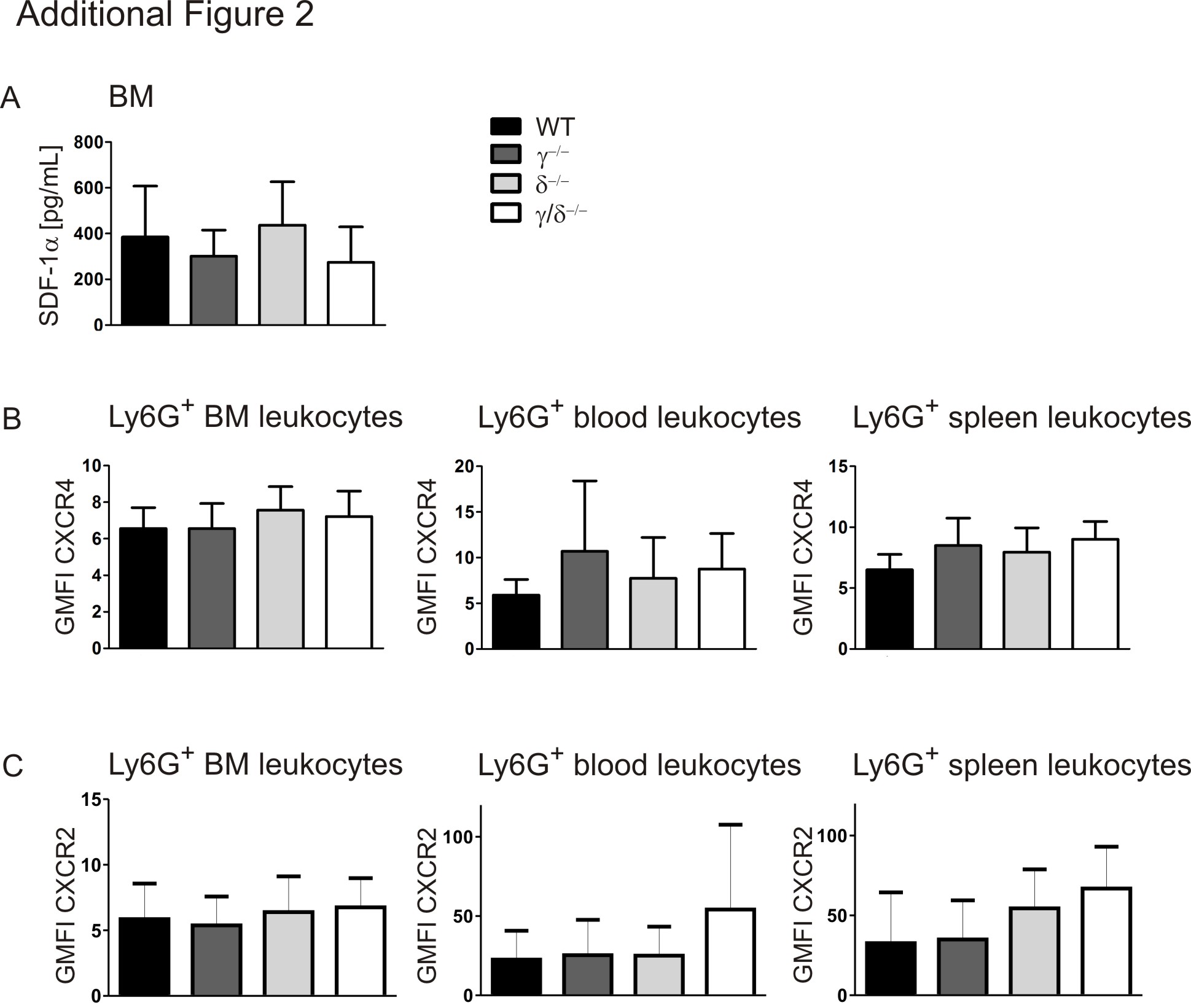

Supplement: Supplementary file 2 — Expression of CXCR4 and CXCL12/SFD-1α in WT, p110γ−/−, p110δ−/−, and p110γ/δ−/− mice. a To measure CXCL12/SDF-1α concentrations in the BM, tibias were flushed with 500 μl PBS, cells were pelleted and supernatants were subsequently subjected to ELISA. Bars represent means + SD of n = 9–10 mice per group. b To determine CXCR4 expression leukocyte suspensions were labeled with fluorescent antibodies and analyzed by flow cytometry. Neutrophils were gated as singlet, live CD3ε − CD19− CD11b+ Siglec-F− Ly6G+ cells and were analyzed for the expression of CXCR4 (CD184). Shown are GMFI of CD184-APC of gated neutrophils in BM (left), blood (middle) and spleen (right). Bars represent means + SD of n = 5–8 mice per group. c To determine CXCR2 expression leukocyte suspensions were labeled with fluorescent antibodies and analyzed by flow cytometry. Neutrophils were gated as singlet, live CD3ε − CD19− CD11b+ Siglec-F− Ly6G+ cells and were analyzed for the expression of CXCR2 (CD182). Shown are GMFI of CD182-APC of gated neutrophils in BM (left), blood (middle) and spleen (right). Bars represent means + SD of n = 7–8 mice per group. (JPEG 248 kb) [file 12964_2017_185_MOESM2_ESM.jpg]

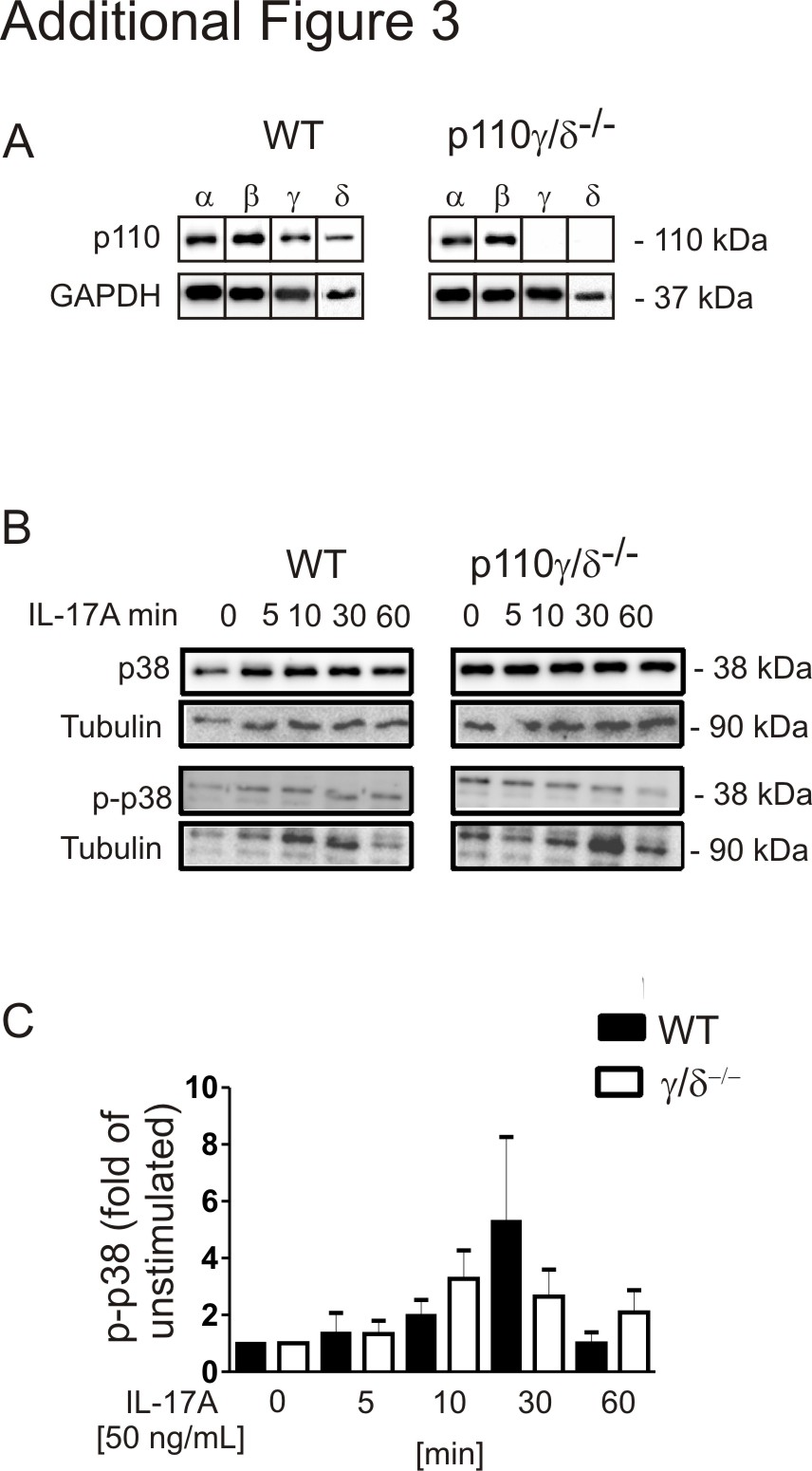

Supplement: Supplementary file 3 — Protein expression of p110α, p110β, p110γ, and p110δ isoforms in primary lung tissue cells from WT and p110γ/δ−/− mice. a Analysis of p110 protein expression in cultured tissue cells from lungs of WT and p110γ/δ−/− mice was performed by immunoblot analysis using anti-p110α, anti-p110β, anti-p110γ, and anti-p110δ specific antibodies. Re-probing for GAPDH served to confirm equal protein loading. b Phospho-p38 expression in lung tissue cells from WT and p110γ/δ−/− mice at different time points following IL-17A (50 ng/ml) stimulation. The blot shows the expression of phospho-p38 and total p38. Re-probing for tubulin served to confirm equal protein loading. c Depicted is a statistical evaluation of phospho-p38 levels. Bars present the average fold change + SD of phospho-p38 levels of unstimulated cells. Phospho-p38 and p38 were first normalized to Tubulin to control for protein loading differences and then phospho-p38 was normalized to p38 levels. Bars represent means + SD of pooled mean values from three independent experiments. (JPEG 151 kb) [file 12964_2017_185_MOESM3_ESM.jpg]

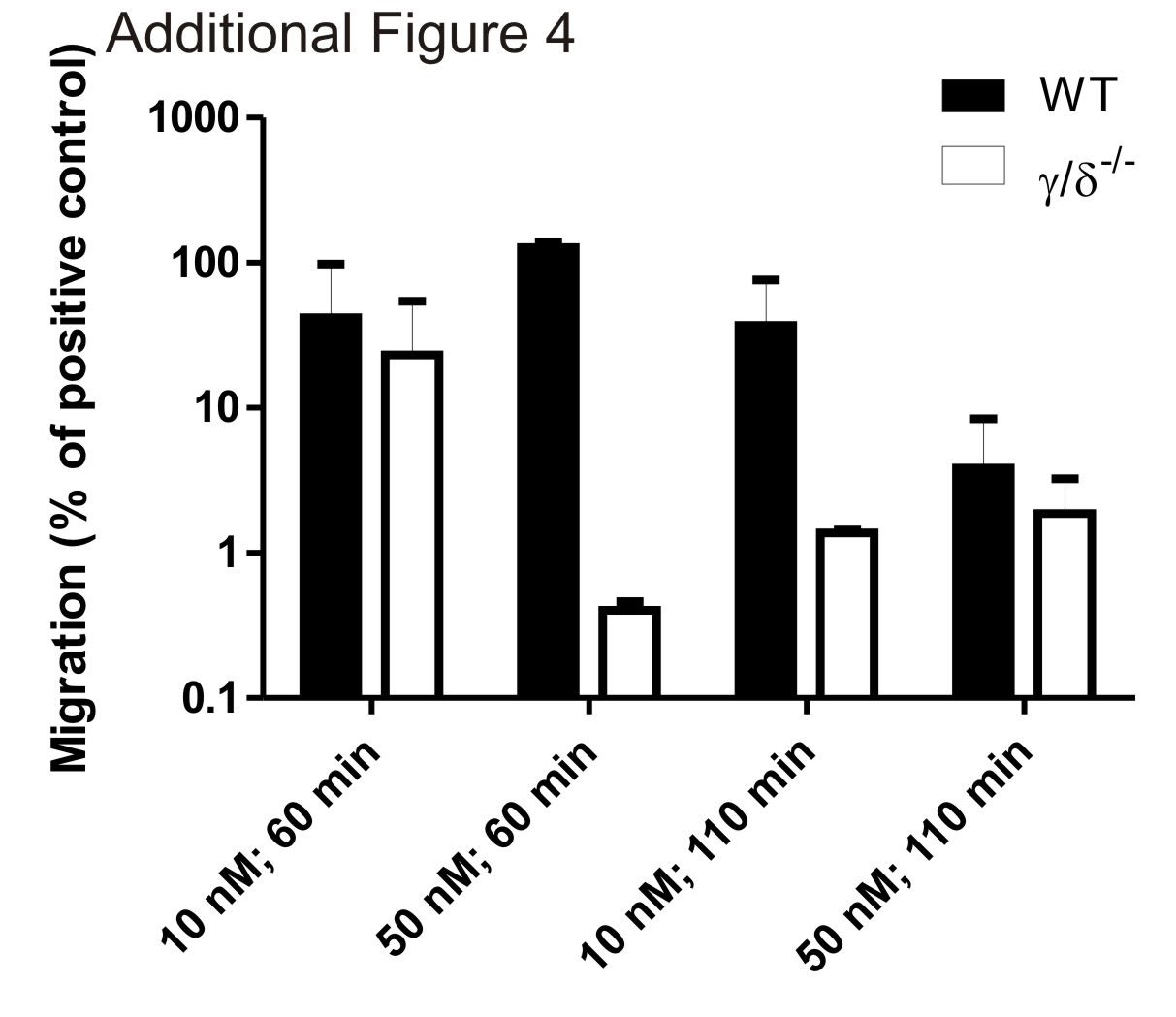

Supplement: Supplementary file 4 — Migration of neutrophils from WT and p110−/− mice upon CXCL12/SDF-1. Transwells inserts (3.0 μm pore size, Costar) were placed in 24-well plates containing CXCL12/SDF-1(10 nM or 50 nM). BM cells labeled with 40 Ly6G-APC were seeded on the upper chamber of each well at 3 × 10^6^ cells per well. In parallel, cells were directly seeded into wells without transwell inserts and served as positive controls (100% migration). Cells were incubated for 60 or 110 min. Then Ly6G+ neutrophils in the lower chambers were counted by flow cytometry. The number of migrated neutrophils is expressed as % of positive control. Bars present means + SD from one experiment measured in duplicates. (JPEG 112 kb) [file 12964_2017_185_MOESM4_ESM.jpg]
